# An Alternative Conformation of the T-Cell Receptor α Constant Region

**DOI:** 10.1016/j.jmb.2010.05.053

**Published:** 2010-07-23

**Authors:** Gijs I. van Boxel, Samantha Holmes, Lars Fugger, E. Yvonne Jones

**Affiliations:** 1 Division of Structural Biology, Wellcome Trust Centre for Human Genetics, The University of Oxford, Roosevelt Drive, Oxford OX3 7BN, UK; 2 Department of Clinical Immunology, Aarhus University Hospital, Skejby Sygehus, DK-8200 N Aarhus, Denmark; 3 MRC Human Immunology Unit, Weatherall Institute of Molecular Medicine, John Radcliffe Hospital, The University of Oxford, Oxford OX3 9DS, UK

**Keywords:** TcR, T-cell receptor, MHC, major histocompatibility complex, pMHC, peptide–MHC complex, MBP, myelin basic protein, CDR, complementarity-determining region, PDB, Protein Data Bank, T-cell receptor, conformational change, crystal structure, signaling, metamorphic proteins

## Abstract

αβ T-cell receptors (TcRs) play a central role in cellular immune response. They are members of the Ig superfamily, with extracellular regions of the α and β chains each comprising a V-type domain and a C-type domain. We have determined the ectodomain structure of an αβ TcR, which recognizes the autoantigen myelin basic protein. The 2.0-Å-resolution structure reveals canonical main-chain conformations for the V^α^, V^β^, and C^β^ domains, but the C^α^ domain exhibits a main-chain conformation remarkably different from those previously reported for TcR crystal structures. The global IgC-like fold is maintained, but a piston-like rearrangement between BC and DE β-turns results in β-strand slippage. This substantial conformational change may represent a signaling intermediate. Our structure is the first example for the Ig fold of the increasingly recognized concept of “metamorphic proteins.”

## Introduction

CD8^+^ and CD4^+^ T cells detect peptide antigens, presented by major histocompatibility complex (MHC; class I or class II) molecules, through αβ T-cell receptors (TcRs) on their cell surface. The extracellular region of αβ TcRs comprises an α chain and a β chain, each consisting of Ig-like variable and constant domains. The TcR C^α^ domain is notable in differing from the classical IgC1 domain.^1–3^ Complementarity-determining loops of the variable domain are responsible for specific binding to peptide antigens upon recognition of MHC molecules. The signal resulting from this recognition event is transduced by CD3γɛ, CD3δɛ, and CD3ζζ. There is substantial evidence from a variety of functional studies indicating that the constant domains of the TcR interface with CD3.^4–9^ Mutagenesis has demonstrated that the interactions of CD3δɛ and CD3γɛ subunits with the TcR C^α^ and C^β^ domains, respectively, contribute to the stability and function of the TcR–CD3 signaling complex.^10^ A series of experiments from Gil *et al.* suggests that TcR triggering requires the TcR–CD3 interface to accommodate large changes in the relative positions of the TcR and CD3 components.^11,12^

Our studies of TcRs specific for a variety of peptide–MHC complexes (pMHCs) have allowed us to sample TcR constant domain structures in a range of crystal lattice environments.^13–15^ This has provided us with the opportunity to survey variation in TcR constant domain structure. Here we present a 2.0-Å-resolution crystal structure of the extracellular domains of a TcR (1F1E8hu) that recognizes the human leukocyte antigen DR2b molecule in complex with a myelin basic protein (MBP) antigen. Comparisons of this unliganded TcR structure with those previously reported for MHC-class-I-restricted and MHC-class-II-restricted TcRs reveal a novel structural rearrangement for the α-chain constant domain. The high-resolution crystal structure of 1F1E8hu provides the first structural evidence that the TcR α-constant domain can adopt two very different but stable conformations. This example of “β-strand slippage” is indicative of structural malleability in the TcR C^α^ domain, which may be of functional relevance.

## Results

The 1F1E8 TcR, cloned from a humanized transgenic mouse,^16^ recognizes the autoantigen MBP85–99 (MBP residues 85–99) presented by human leukocyte antigen DR2b with the same fine specificity as cognate human TcRs.^17^ To facilitate protein production and crystallization of a soluble form of 1F1E8, we exchanged its mouse constant domains with those of a human TcR (JM22) that has consistently provided high-resolution structures.^13,15^ The linker regions between TcR variable and constant domains in mouse and human are relatively conserved, and formation of the chimeric 1F1E8hu TcR introduces no residue substitutions that are incompatible with structural integrity. Biophysical experiments demonstrated that the humanized 1F1E8hu TcR recognizes the DR2b–MBP85–99 complex.^18^ In transgenic mice, we have previously shown that replacing the human TcR constant domains with those of the mouse receptor does not alter the TcR recognition of the DR2b–MBP85–99 complex.^16^

**Fig. 1:**
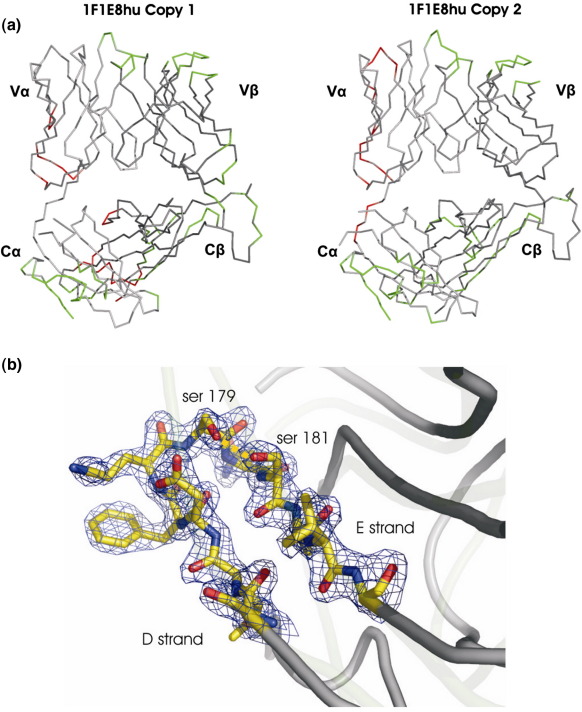
Crystal contacts in the 1F1E8hu structure. (a) Both copies of the 1F1E8hu TcR are shown. The α chain is shown in light gray, and the β chain is shown in dark gray. Residues involved in crystal contacts within the crystallographic asymmetric unit (asu) are shown in red, and contacts between molecules in different asu's are depicted in green. (b) Overlay of the C^α^ domains of 1F1E8hu (dark gray) and JM22 (light gray), electron density at 1σ illustrating the shortened DE loop in 1F1E8hu, and residues 174–184 depicted in ball-and-stick. Carbons are highlighted in yellow, oxygens are highlighted in red, and nitrogens are highlighted in blue. Cartoons were produced using PyMOL (http://www.pymol.org).

The structure of 1F1E8hu was determined by molecular replacement and refined to a crystallographic *R*-value of 24% (*R*_free _= 20%) using data up to 2.0 Å resolution (Table 1). There are two copies of the molecule per crystallographic asymmetric unit (Fig. 1a); for both of these copies, there is clear electron density (representative electron density is shown in Fig. 1b) for the variable and constant regions of the α and β chains. The two copies of 1F1E8hu are identical in structure (RMSD of 0.3 Å on 100% equivalent α-carbons); copy 1 is used in the following analysis (Fig. 1).

### Variable domains

Both the α variable domain and the β variable domain are similar to those in previously reported TcR structures, with the α and β complementarity-determining region (CDR) 1 and CDR2 loops adopting canonical main-chain conformations.^19^ In analyzing the variable domains, we were particularly interested in the crystal structures of two previously reported TcRs: Ob1A12^20^ and B7.^21^ 1F1E8hu recognizes the same antigen as Ob1A12 (a TcR isolated from a multiple sclerosis patient with a relapsing–remitting disease course; the Ob–MBP–DR2b complex structure shows a distinctive TcR binding mode positioned over the N-terminal portion of the MBP peptide) and the human TcR B7, which has a relatively high sequence identity to 1F1E8hu (59% and 56% for V^α^ and V^β^, respectively) and typifies foreign antigen-reactive TcRs in its pMHC-binding characteristics. Structural superpositions of 1F1E8hu on B7 and Ob1A12, respectively, give RMSDs between C^α^ equivalents of 1.2 Å and 0.9 Å for the V^α^ domain, and C^α^ equivalents of 1.0 Å and 0.9 Å for the V^β^ domain. The buried surface between the 1F1E8hu V^α^ domain and the 1F1E8hu V^β^ domain is 1486 Å^2^, which is very similar to those of other TcRs (1450 Å^2^ in B7 and 1498 Å^2^ in Ob1A12).

**Fig. 2:**
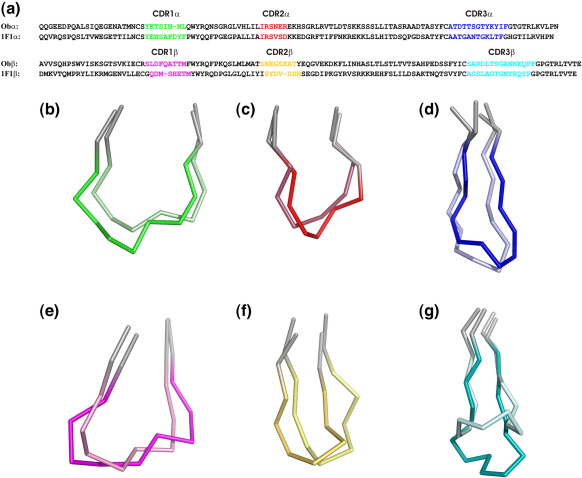
Comparison of 1F1E8hu and Ob1A12 TcR CDR loops. (a) Sequence alignment of Ob1A12 and 1F1E8hu TcR V domains with CDR loops indicated. (b–g) Cα traces of Ob1A12 and 1F1E8hu TcR CDR1α (b), CDR1β (c), CDR2α (d), CDR2β (e), CDR3α (f), and CDR3β (g) loops. The darker loop of each color is 1F1E8hu, and the lighter loop of each color is Ob1A12. The Ob1A12 loops are taken from Hahn *et al.*^20^ Superpositions were made using *SHP*,^22^ and PyMOL was used to generate the figure.

Since Ob1A12 and 1F1E8hu recognize the same pMHC, we carried out a detailed comparison of the six CDR loops, which determine the binding characteristics of each TcR (Fig. 2b). The number and nature of many of the amino acids in the CDR loops vary considerably between 1F1E8hu and Ob (Fig. 2a). Main-chain conformations of the TcR α-chain and β-chain CDR1 and CDR2 loops generally correspond to one of three or four canonical structures defined by conserved residue motifs.^19^ The CDR1α, CDR2α, CDR1β, and CDR2β loops of 1F1E8hu each conform to one of these canonical structures, whereas all four of the equivalent loops of Ob1A12 do not. These differences in amino acid usage and main-chain conformation introduce profound differences in the shape and electrostatic footprint of the 1F1 TcR relative to those of Ob1A12, consistent with the different recognition characteristics of these two TcRs observed for serial alanine substitution of the MBP peptide in T-cell stimulation assays.^17,18,23,24^

### Constant domains

**Fig. 3:**
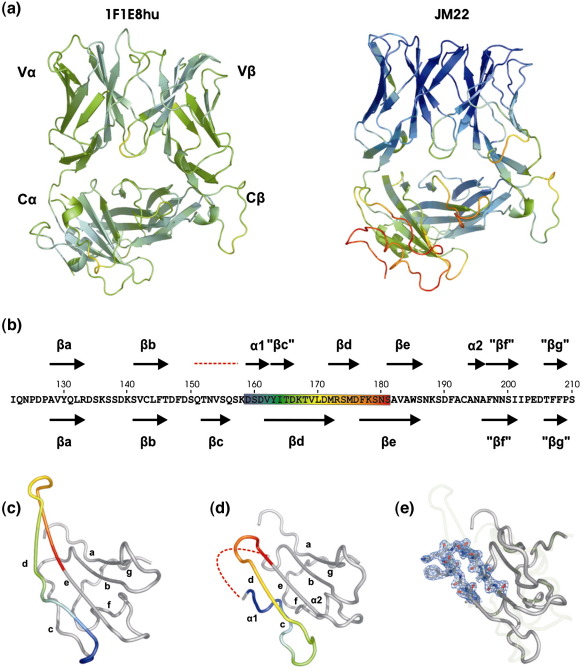
Structural comparison of 1F1E8hu and JM22 TcRs. (a) Ribbon representation of the 1F1E8hu and JM22 structures colored according to the *B*-factor, where dark blue is the lowest (15 Å^2^) and deep red is the highest (75 Å^2^). (b) Amino acid sequence for the human TcR α-chain constant domain. β-Strands are represented by arrows. The red broken line indicates the disordered region in 1F1E8hu. Residues 159–181 are rainbow colored, reflecting equivalent residues in the C^α^ domains of JM22 (c) and 1F1E8hu (d). (e) Overlay of the C^α^ domains of 1F1E8hu (dark gray) and JM22 (light gray), and electron density at 1σ illustrating the shortened DE loop in 1F1E8. Cartoons were produced using PyMOL.

**Fig. 4:**
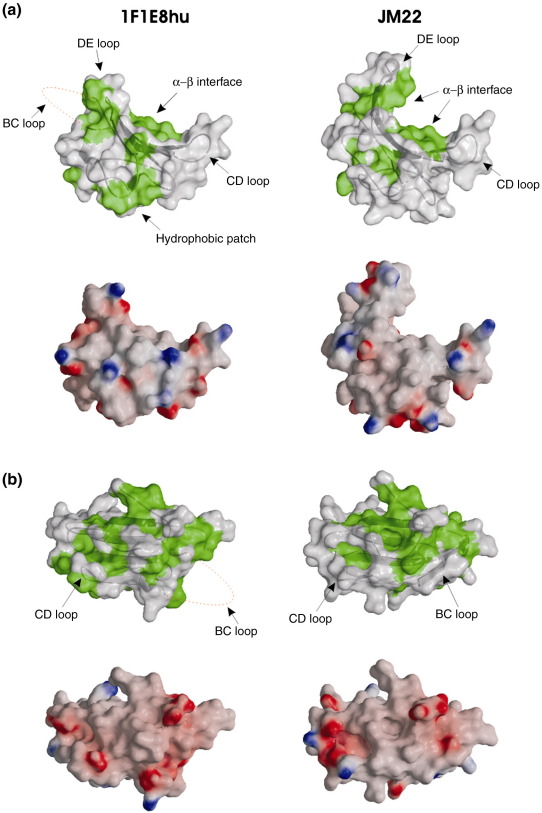
Surface representations of the 1F1E8hu and JM22 C^α^ domains. (a) C^α^ domains of 1F1E8hu and JM22 are shown in identical orientations. Green surfaces represent exposed hydrophobic residues. Electrostatic surface potentials are shown in red for negative potentials (− 20 kT) and in blue for positive potentials (+ 20 kT). (b) Rotation of (a) showing the top sheet of the C^α^ domain. Surfaces were calculated using the program GRASP (http://trantor.bioc.columbia.edu/grasp/).

The main-chain atoms of the β-chain constant domain (C^β^) are superimposable on those of JM22, which has a 100% sequence homology for the constant domains [Protein Data Bank (PDB) code 1OGA],^13^ with the exception of minor changes in one β-strand (discussed in the text below). However, the α-chain constant domain (C^α^) shows a large structural deviation from all previously reported structures, including the identical sequence in JM22 (Fig. 3a–c). In these published structures, the C^α^ domain adopts an unusual IgC-like fold. β-Strands “A,” “B,” “E,” and “D” form one sheet (bottom sheet) of the C^α^ domain, as in a conventional IgC domain, but strands “C,” “F,” and “G” (top sheet), although linked to the bottom sheet by a disulfide bond, pack only loosely against it, leading to surface exposure of some hydrophobic core residues^25^ (Fig. 3c). In 1F1E8hu, the main-chain conformation of strands A and B and the AB loop (residues 116–150) matches that of JM22 and all other TcR structures; however, at the BC loop, the sequence register between 1F1E8hu and JM22 shifts. Usually, the BC loop is 4 residues long; in the 1F1E8hu structure, 11 residues contribute to a significantly larger but flexible loop [of which residues 151–157 (QTNVSQS) are disordered]. Although mobile, this region will occupy a significantly larger volume at the domain surface than the standard BC loop (Fig. 3d). The residues forming strand C in 1F1E8hu therefore differ from those in all other TcR structures (e.g., JM22) (Fig. 3b); the sequence is effectively displaced towards the N-terminus. Although the C strand in JM22 is not involved in a classical acceptor–donor hydrogen-bond network, it adopts an extended β-strand-like main-chain conformation; the equivalent secondary structural element in 1F1E8hu starts as a short 3_10_ helical turn, followed by an extended chain. The CD loop is different in shape, length, and, most interestingly, charge distribution (there is an extensive hydrophobic patch on the 1F1E8hu surface) due to the shift in residue usage (Fig. 3a). Remarkably, the main-chain positions of the D strand in 1F1E8hu and JM22 are similar, even though the residues that form this part of the structure differ [residues 172–176 (MRSMD) in 1F1E8hu; residues 164–168 (ITDKT) in JM22]. The replacement of Ile164 by Arg173 at the start of strand D contributes to the different CD loop conformation. The main-chain and side-chain atoms of residues Ser174 (Thr165), Met175 (Lys166), and Asp176 (Thr167) adopt similar positions in both structures, with no obvious distorting effect on neighboring structural elements. The D strand ends prematurely in 1F1E8hu compared to JM22; a hydrogen bond between the side-chain hydroxyl group of Ser179 and the side-chain hydroxyl group of Ser181 stabilizes the shortened DE loop (Figs. 1b and 3e). Interestingly, equivalent serine residues (residues 169 and 171) of the γδ TcR form a similar hydrogen bond in the connective DE loop.^26,27^ In the JM22 structure, these serine residues are some 6 Å apart in strand D, allowing it to continue for another four residues before forming a β-hairpin turn into strand E. An omit map of this region of 1F1E8hu shows well-defined density for all main-chain and side-chains atoms, confirming the novel arrangement of the amino acid residues (Figs. 1b and 3e). The truncation of the β-hairpin in 1F1E8hu further affects the shape and charge distribution of the TcR surface. In JM22, an extra nine residues are used to form the DE β-hairpin; short circuiting this loop allows the residues of 1F1E8hu to get back in register with those in JM22 at the point where the E strand of 1F1E8hu starts at Asn180. From Ser181 onwards, the residue usage in 1F1E8hu and JM22 is conserved, but variations in main-chain and side-chain conformations result in significant changes in surface characteristics (Fig. 4).

**Fig. 5:**
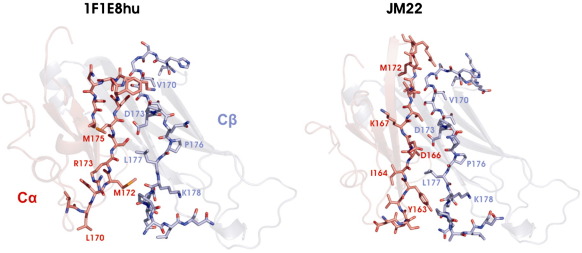
α–β interface of the 1F1E8hu and JM22 constant domains. The C^α^ domain is shown in red, and the C^β^ domain is shown in blue. Strand D of the C^α^ domain and strand D of the C^β^ domain are represented in ball-and-stick atoms; the rest of the protein is shown in cartoon form.

Overall, the structure of the 1F1E8hu C^α^ domain maintains a global IgC-like scaffold, but reveals a piston-like conformational change; from the BC turn to the DE turn, the residue positions are completely different, with many of those normally forming strands becoming involved in interstrand loops and *vice versa* (Fig. 3b–d). The C^α^ domain contains several N-linked glycosylation sites, but none lies at positions that could impede this structural rearrangement. Although the core topology of the 1F1E8hu C^α^ domain remains similar to that of JM22 (RMSD of 2.3 Å for 78% α-carbon pairs), comparison using the program GAP (which evaluates the relative position of chemically identical residues in 1F1E8hu and JM22; J. Grimes and D. I. Stuart, unpublished program) reveals an RMSD of 8.7 Å for equivalent residues. A very different set of residues now contributes to the interface with the C^β^ domain, yet the hydrophobic character required for this interaction^25^ is maintained (Fig. 4a). It has been previously been noted that the C^β^ domain must be able to interface with at least two different surfaces: the pre-TcR α subunit^4^ and the TcR α chain. Comparison of 1F1E8hu and JM22 shows that C^α^ interface changes are accommodated by relatively small structural adjustments in the D strand of the C^β^ domain (Fig. 5), a region already seen to have significant flexibility (as judged by crystallographic *B*-factors) in previous structures (Fig. 3a).

The C^α^ domain has historically been the most disordered region of the TcR, suggesting a tendency to adopt different conformations. For example, the C^α^ domains were omitted from the final models of several TcR–pMHC structures due to lack of electron density,^28,29^ and the initial structure determined for the isolated murine 2C TcR was devoid of any clear electron density for the C^α^ domain.^25^ These examples of C^α^ domain disorder contrast with the relatively well-ordered electron density in the tightly abutting C^β^ domains and suggest that some crystals may have sampled more than one C^α^ domain conformation. For the murine 2C TcR, a single domain conformation was subsequently stabilized by crystallization in a different space group.^25^ Analysis of the average *B*-factor for each domain of all TcRs solved to date confirms that the C^α^ domain, when visualized, has the greatest mobility (Table 1). This flexibility is particularly pronounced in the top sheet (Fig. 3a). In both crystallographic copies of the 1F1E8hu structure, the C^α^ top sheet makes contact with neighboring molecules in the crystal (Fig. 1a) and is well ordered with an average *B*-factor of 36.4 Å^2^ (the overall *B*-factor for 1F1E8hu is 35.8 Å^2^).

## Discussion

Several distinctive features of the TcR C^α^ domain may facilitate the “β-strand slippage” that we observed in the 1F1E8hu structure. Firstly, the number of amino acids between anchoring cysteine residues is only 50, as opposed to 60–65 in the classical IgC domain. Secondly, the otherwise conserved Trp15 residue upstream of the first cysteine is omitted in the TcR C^α^ domain. These deviations from the normal IgC domain produce a more loosely packed core, allowing for structural rearrangement within the domain.^25^

In line with our findings, other groups have reported similarly large conformational changes in a range of proteins: firstly, the crystal structure of a metastable dimeric form of the variable domain from the cell surface adhesion molecule CD2.^30^ The dimer results from segments of two polypeptide chains intercalating to form two domains, each of which remains similar to the conventional variable domain fold. For the 1F1E8hu structure, there is no such intercalation of domains, but rather β-strand slippage. This phenomenon resembles the observation that ARNT (*a*ryl hydrocarbon *r*eceptor *n*uclear *t*ranslocator) PAS-B (*P*er–*A*rnt–*S*im *B*) can adopt an alternative β-sheet register.^31^ The β-sheet surface in ARNT PAS-B mediates protein–protein interactions, and the authors speculate that the structural malleability indicated by this β-strand slippage maybe related to the flexibility required for binding to several protein partners. Similarly, the spindle assembly checkpoint protein Mad2 can adopt either of two distinct conformations while maintaining a large common substructure.^32^ More drastically, the protein lymphotactin can adopt two distinct folds in equilibrium to facilitate multiple functional states.^33^ Our observation that the 1F1E8hu C^α^ domain is intrinsically able to adopt two conformational states raises the question as to what role this structural plasticity plays in TcR function. One very appealing theory is that flexibility and/or conformational changes in the TcR C^α^ domain could provide a mechanism to allow it to adapt to binding CD3 and hence play its role in TcR signaling.^25,34,35^

Experiments by other groups support the hypothesis that the C^α^ domain could play a vital role in TcR signal transduction. Kuhns and Davis have shown, by mutagenesis of different C^α^ loops, that the CD3 contact region is likely to be located within the DE loop.^10^ It is further noteworthy that residues affected by the altered conformation of 1F1E8hu have previously been identified as making crucial contacts with CD3; the antibody H28-710 (which binds C^α^ domain residues 150–177) prevents TcR–CD3 interaction,^5^ and the association of CD3ζ with TcR αβγδɛ is completely abolished by a single-point mutation (phenylalanine to valine at position 195) in the C^α^ domain.^6^ Gil *et al.* showed that, upon antigen recognition, the CD3 complex undergoes a conformational change to initiate T-cell signaling,^12^ and they postulated that this change could be propagated through the TcR. Hence, any structural rearrangement in the C^α^ domain may be transmitted to CD3 and *vice versa*. Thus, our data on the structural plasticity of the C^α^ domain are of direct relevance to a number of hypotheses that have proposed that a structural rearrangement in the TcR–CD3 complex is necessary in early T-cell signaling.^36^

The ability of certain proteins to adopt multiple conformations is central to many biological processes, and the increasing number of examples of this phenomenon has recently been characterized by the term “metamorphic proteins.”^37^ The structure of 1F1E8hu reveals that the TcR C^α^ domain is also able to adopt two very different stable conformations, a fundamental property that is potentially central to TcR function.

## Methods

### Protein expression, purification, and biophysical characterization

The TcR V^α^ and V^β^ regions were PCR amplified from cDNA derived from the mouse 1F1E8 T-cell hybridoma. These regions were then spliced by overlap extension to human TcR C^α^ and C^β^ regions, including C-terminal extensions designed to assist the formation of an interchain disulfide bridge (as previously reported for the structure of JM22).^13,18^ PCR products for these α and β chains were digested with NdeI and XhoI, cloned into pET-22b(+) (Novagen), and separately expressed in *Escherichia coli* cells (B834 for α chain and B834pRareLysS for β chain; Novagen). The heterodimeric functional TcR (termed 1F1E8hu) was produced using previously described protocols.^13^ In brief, the inclusion bodies resulting from the expression of the TcR α and β chains were mixed and dialyzed in the presence of a refolding buffer containing a redox couple, and refolded protein was then purified by column chromatography (anion exchange followed by gel filtration).

Prior to crystallization, the refolded protein sample was subjected to quality control by means of SDS-PAGE, mass spectrometry, and dynamic light scattering. Because the correctly refolded protein contains an interchain disulfide bond, all these techniques were performed under reducing and nonreducing conditions by the addition or omission of β-mercaptoethanol from the sample. The predicted molecular mass of the correctly refolded protein is 54,423 Da. SDS-PAGE analysis of the purified sample clearly showed the presence of a single 53-kDa band, which, upon the addition of reducing agent, ran as two distinctly separate bands of ∼ 26 kDa and ∼ 29 kDa (with similar intensities), concurrent with the 1F1E8hu α and β chains, respectively. Mass spectrometry further verified the presence of correctly refolded heterodimeric TcR; the nonreduced sample had a molecular mass of 54,416 Da and, under reducing conditions, the presence of equimolar amounts of α chain (25,598 Da) and β chain (28,825 Da) with the expected molecular masses was confirmed. Dynamic light-scattering experiments were performed on the nonreduced sample to ensure that no aggregation of the protein sample had occurred; this technique, although not as sensitive, confirmed a homogeneous solution containing nonaggregated protein with a calculated mass very similar to that of 1F1E8hu.

### Crystallization and structure determination

Crystallization trials were carried out using previously reported robotic technologies and protocols.^15,29^ 1F1E8hu was crystallized by vapor diffusion at 10 mg/ml using 200-nl sitting drops at 20 °C. The precipitant solution contained 0.3 mM (NH_4_)_2_ hydrogen citrate and 20% wt/vol polyethylene glycol 3000. The resulting crystals were plate-like with dimensions of 100 μm × 50 μm × 10 μm. The crystals were cryoprotected in mother liquor containing 20% glycerol and cooled to ∼ 100 K. X-ray data were collected at the European Synchrotron Radiation Facility (Grenoble, France) on beamline id14-EH1 and processed using the program package HKL2000.^38^ A molecular replacement solution was readily found using an MHC-class-I-restricted TcR (PDB code 1BD2) as initial search model^21^ in Phaser.^39^ The structure was built using O,^40^ refined with REFMAC,^41^ and checked using the program PROCHECK.^42^ Two copies of the 1F1E8hu heterodimer were present in the crystallographic asymmetric unit, designated as copies 1 and 2. Superposition of copies 1 and 2 showed that they are near identical (RMSD of 0.3 Å on 100% equivalent α-carbons), with only a few differences in side-chain positions exposed to the solvent. For this reason, copy 1 was used for the calculations and figures in this work, unless otherwise stated.

### Accession numbers

Coordinates and structure factors have been deposited in the PDB under accession number 3MFF.

## Figures and Tables

**Table 1:** Data collection and refinement statistics for X-ray crystallography

*Data collection statistics*^a^			
Resolution (Å)	30–2.0 (2.05–2.00)		
Completeness (%)	91.7 (63.9)		
Multiplicity	2.7 (1.9)		
*I*/σ*I*	20.2 (3.0)		
Number of observations/unique reflections	690,731/74,855		
*R*_sym_^b^	5.1 (25.9)		

*Refinement statistics*			
Wilson *B*/average *B*	29.5/36.0		
Number of non-H atoms/waters	6958/802		
Number of reflections	70,789 (3611)		
RMSD^c^ bonds/angles	0.014/1.5		
Ramachandran^d^	89.2 9.6 0.5 0.7		
*R*_cryst_/*R*_free_^e^ (%)	20.0/24.3		


	*B*-factor (Å^2^)	*B*-factor of equivalent domain in JM22 (Å^2^)	RMSD with JM22 (% α-carbon residues used)
1F1E8hu	35.8	34.4	2.1 (90)
1F1E8hu V^α^	35.5	28.0	1.5 (96)
1F1E8hu V^β^	36.2	27.3	1.1 (98)
1F1E8hu C^α^	36.4	52.5	2.3 (78)
1F1E8hu C^β^	35.4	37.0	0.6 (99)

RMSDs were calculated using the program *SHP* (D. I. Stuart, unpublished).

a Data in parenthesis are for the highest-resolution shell.

b *R*_sym_ = σ_*j*_|〈*I*〉 − *I_j_*|/σ〈*I*〉 where *I*_*j*_ is the intensity of the *j*th reflection, and 〈*I*〉 is the average intensity.

c Root-mean-square deviation from ideal values.

d Values are expressed as the percentage of amino acids in the “core,” “allowed,” “generously allowed,” and “disallowed” regions, respectively.

e Five percent of data have been set aside for cross-validation calculations.
